# Quality assurance of malaria rapid diagnostic tests used for routine patient care in rural Tanzania: microscopy *versus* real-time polymerase chain reaction

**DOI:** 10.1186/s12936-015-0597-3

**Published:** 2015-02-19

**Authors:** Irene M Masanja, Meredith L McMorrow, Mussa B Maganga, Debora Sumari, Venkatachalam Udhayakumar, Peter D McElroy, S Patrick Kachur, Naomi W Lucchi

**Affiliations:** Health System Sciences, Ifakara Health Institute, PO Box 78373, Dar es Salaam, Tanzania; Malaria Branch, Centers for Global Health, US Centers for Disease Control and Prevention, Atlanta, GA USA; US Public Health Services, Rockville, Maryland USA; US President Malaria Initiative (PMI), Dar es Salaam, United Republic of Tanzania

**Keywords:** Rapid diagnostic test (RDT), Microscopy, qPCR, Quality assurance (QA), Routine malaria tests

## Abstract

**Background:**

The World Health Organization (WHO) recommends parasitologic confirmation of suspected malaria cases before treatment. Due to the limited availability of quality microscopy services, this recommendation has become scalable following increased use of antigen-detecting malaria rapid diagnostic tests (RDTs) in many malaria-endemic countries. This study was carried out to monitor quality of RDT performance in selected health facilities using two quality assurance (QA) methods: reference microscopy and detection of parasite DNA by real-time quantitative polymerase chain reaction (qPCR) on dried blood spots (DBS).

**Methods:**

Blood samples for QA were collected from patients undergoing RDT for diagnostic confirmation of malaria during two to three consecutive days per month in 12 health facilities in rural Tanzania. Stained blood smears (BS) were first examined at the district hospitals (BS1) and then at a reference laboratory (BS2). Discordant BS1 and BS2 results prompted a third examination. Molecular analysis was carried out at the Ifakara Health Institute laboratory in Bagamoyo.

**Results:**

Malaria RDTs had a higher positivity rate (6.5%) than qPCR (4.2%) or microscopy (2.9% for BS1 and 2.5% for BS2). Poor correlation was observed between RDT and BS results: BS1 (K = 0.5), BS2 (K = 0.43) and qPCR (K = 0.45), challenging the utility of these tests for RDT QA. In addition, many challenges related to qPCR processing were recorded and long delays in obtaining QA test results for both microscopy and qPCR.

**Conclusions:**

Overall there was limited agreement among the three diagnostic approaches and neither microscopy nor qPCR appear to be good QA options for RDTs under field conditions.

## Background

The 2012 World Malaria Report identified tremendous achievements toward reduction of the global malaria burden [1]. Success has mainly been attributed to increased coverage of malaria control interventions such as use of insecticide-treated mosquito bed-nets (ITNs), indoor residual spraying (IRS), and use of effective treatments with artemisinin-based combination therapy (ACT). In 2010, the World Health Organization (WHO) recommended parasitologic confirmation of suspected malaria cases before initiating anti-malarial treatment [2] and many malaria endemic countries in sub-Saharan Africa have adopted this policy [3,4]. The scale-up of antigen-detecting malaria rapid diagnostic tests (RDTs) for Plasmodium species forms a vital part of the strategy to confirm malaria infection prior to treatment in resource-poor settings [5].

Laboratory confirmation of malaria is being achieved, in part, due to the increased use of RDTs. With increased testing, the effect of incorrect test results is becoming a challenge in clinical management of patients suspected to have malaria infection [6,7]. Initial field trials of histidine rich protein (HRP)-2 based RDTs demonstrated a sensitivity and specificity of over 90% for *Plasmodium falciparum* at parasite densities over 200 parasites/μL [8], particularly in laboratory or controlled settings. However, later studies have demonstrated that RDT sensitivity varied greatly between health facilities in Tanzania (18.8% to85.9%), with data difficult to interpret due to poor microscopy slide quality from some facilities [9]. Likewise other studies have reported significant variations in RDT sensitivity and specificity [10-13] and particularly when RDTs are exposed to adverse conditions, such as higher temperature [14].

Among the earlier recommendations of WHO was to assess performance of RDTs through periodic comparison of RDT results to reference microscopy [15]. In this recommendation, each health facility using RDTs was expected to submit blood smears from twenty RDT positive and twenty RDT negative patients monthly for evaluation [15]; however, in the wake of changing malaria transmission patterns this may not be feasible in areas of low transmission, as they may not have 20 positive RDTs in a month. Additionally, challenges of obtaining good quality blood smear results from lower level health facilities for further assessment at reference laboratories have been reported [16-19]. A need for a practical quality assurance (QA) procedure for RDTs was apparent.

Tanzania’s Ministry of Health and Social Welfare through the National Malaria Control Programme (NMCP) began to deploy RDTs in 2010 in selected regions as a way to expand and strengthen malaria diagnostic capacity throughout the country. The NMCP recognized this effort to deploy RDTs would require a suitable QA method that could be adopted nationwide. The Ifakara Health Institute (IHI) in collaboration with the US Centers for Disease Control and Prevention (CDC) Malaria Branch and the U.S. President’s Malaria Initiative (PMI), undertook a study in early 2010 to assess two methods of RDT QA and their timeliness. This approach included comparison of RDT used in routine care of patients, to reference microscopy and to a real-time quantitative PCR (qPCR) assay.

## Methods

Tanzania adopted a phased approach to the scale-up of RDTs and first introduced the tests in three regions: Iringa (low endemicity), Kagera (high endemicity) and Pwani (high endemicity) in 2009. All levels of healthcare facilities (hospital, health center, and dispensary) were targeted to receive RDTs. These three regions accounted for approximately 12.8% of the Tanzania mainland’s estimated 41.9 million population in 2010 [20].

### Study locations

A convenience sample of 12 health facilities with high utilization rates in Iringa Region were selected to participate in this study; six in Mufindi District (Mafinga District Hospital, Kibao Health Center, Usokami Health Center, Malangali Health Center, Igomaa Dispensary and Sadani Dispensary) and six in Iringa Rural District (Tosamaganga Designated District Hospital, Idodi Health Center, Kimande Health Center, Mlowa Dispensary, Ifunda Dispensary and Ilambilole Dispensary). The selection was intended to include facilities in all levels of care: primary (dispensary), secondary (health center) and referral (district hospital).

### Sample collection

RDT services were available for routine clinical care to all study facilities. To perform a test, health worker obtained finger-prick capillary blood specimens from patients suspected to have acute malaria infection. The blood was collected in a transfer device (either a capillary tube or loop) and placed in the appropriate well on the RDT where it is absorbed by the nitrocellulose paper. From the same finger prick an additional 2–3 drops of blood for a thick blood smear (BS) and 2–4 drops of blood for a dried blood spot (DBS) were collected. QA study samples were collected for a two to three day period each month, during the 5 months of data collection.

### Training

Each facility received training on how to appropriately collect, label and store specimens. Health workers at all participating facilities were trained to perform RDTs by the local district council health management team (CHMT) during the national RDT roll out in 2009. In addition, all technicians at each district hospital laboratory had recently undergone a comprehensive microscopy competency training at the National Institute for Medical Research headquarters, Dar es Salaam, under the Walter Reed Army Institute of Research programme [21].

The RDTs used in this evaluation were those available at the government facilities through the Ministry of Health and Social Welfare and included SD Bioline®, ParaHIT® and Paracheck® obtained through the existing national procurement and distribution system. Log books and unique ID stickers were provided to record RDT results and for specimen tracking.

### Supervision, specimen processing, and QA logistics

Study facilities received monthly visits from the district QA supervisor (a laboratory technician). Once a month, two days prior to the arrival of the supervisor, thick BS and DBS were collected from all suspected malaria patients receiving routine RDT at the health facility. A sticker was placed in the log book where RDT results were recorded. At the facility, the QA supervisor collected 40 BS/DBS pairs prepared in the previous two days, selecting pairs at random if greater than 40 were available. BS samples were sent directly to the district laboratory technician, who was blinded to RDT results, to be stained and examined (BS1). At the district laboratory, each BS was stained using 10% Giemsa solution buffered to pH 7.2, and stained for 8–15 minutes. When a dilution of 5% was used, staining was done for 30 minutes.

All BS together with the corresponding DBS were sent to the IHI Bagamoyo laboratory for examination by a second microscopist (BS2), blinded to RDT and BS1 results. For both BS1 and BS2, a blood smear was declared negative after reading 100 high power fields. Parasites were counted in reference to 200 white blood cells. Any discordant results between BS1 and BS2 were resolved by a third microscopist (BS3), a senior technician from the Muhimbili University of Health and Allied Sciences (MUHAS).

### qPCR assays

DBS samples were processed and analysed using qPCR in the IHI’s research laboratory located in Bagamoyo, Pwani (qPCR1). As a validation, a subset of samples was retested at CDC, Atlanta, GA (qPCR2). DNA was extracted from DBS samples using a QIAamp DNA Mini Kit (QIAGEN, Valencia, CA (Qiagen method)). The DNA was aliquoted and stored at −20°C until used. For quantification, a 10-fold serial dilution of *P. falciparum* (3D7) was prepared at the Malaria Branch laboratory, CDC, Atlanta, GA and sent to IHI, Bagamoyo. Briefly, parasites were cultured on an orbital shaker to facilitate one parasite per red blood cell infection. The culture was synchronized by the sorbitol method to select for ring stage parasites which can reproducibly be used for quantification of DNA. The parasite density in parasites/μL was determined. Ten-fold dilutions of the parasite culture were prepared with parasite densities ranging from 200,000 parasites/μL to 20 parasites/μL. DNA was extracted from each dilution using the QIAamp DNA Mini Kit. All qPCR assays were performed using commercially available PCR buffer and taq polymerase (New England Biolabs, Ipswich, MA). Primers and a HEX-labeled Taqman probe to the *P. falciparum* beta-tubulin gene were designed and used as previously described [22]. DNA amplification was carried out following the cycling conditions: 95°C for 5 minutes, followed by 50 cycles of 95°C for 20 seconds and 58°C for 1 minute using the Mx3005p real-time PCR machine (Agilent technologies). The serially diluted 3D7 DNA standard was run in each experiment and was used to estimate the parasite density in the field samples. All samples were tested in triplicate. A thousand DNA samples were sent to CDC from which about 300 samples were systematically selected by picking every 3rd sample. These were processed by qPCR as described above and results used to validate the qPCR1 results from Bagamoyo.

### Data processing and analysis

Data were entered in Microsoft Access (Microsoft Corporation, Microsoft Way- Redmond, Washington) and analyzed in STATA 11 (StataCorp, College Station, Texas). Only samples with a parasite density of 200 parasites/μL as determined by qPCR1, were considered as positives in the comparison analysis, as this is considered the potential limit of detection for RDTs [23]. Samples with negative qPCR1 results were included as negatives and samples with low-density infection (<200 parasites/μL) by qPCR1 were excluded from analysis. Proportions of positive tests were compared across the two districts and against the three diagnostic methods using a chi-square test. Parasite densities from microscopy readings (BS1 and BS2, or BS1 and BS2/ BS3) were averaged to get mean variable for microscopy results (here referred to as BSsum). Chi-squared test assessed differences in proportion of positive tests by district. Cross tabulations between tests results were used to estimate each method’s sensitivity, specificity, positive and negative predictive values for RDT, taking BSsum and qPCR1 as gold standard. McNemar’s test was used to assess the difference in proportion of positive results by type of test. Kappa statistic was used to quantify the strength of the tests’ results agreement.

### Ethical clearance

The study protocol was determined to be exempt from ethical review by CDC and received IHI ethical approval number IHI/IRB/No. A 73–2009 as well as a national ethical permit from the Tanzanian National Institute for Medical Research: NIMR/HQ/R.8a/Vol. IX/1534. The sample transportation (import/ export) permit certificate No. 00007867, dated September 1st, 2010, was issued by the Tanzania Private Health Laboratories Board to allow movement of DNA aliquots to CDC laboratory in Atlanta, GA, USA.

Verbal agreements to use patients’ level data for this analysis were acquired at the district health management level when permission was granted to conduct this study in the selected health facilities. No personal identifiers were used for patient’s data.

## Results

A total of 2,369 samples were collected from patients who attended participating health facilities and 2,324 (98%) samples had complete information. During data entry, 487 (20.9%) BS and/or DBS samples were found to have duplicate IDs due to a printing error of specimen labels. In addition, BS and DBS samples from 129 and 333 participants, respectively, were not collected which further reduced the number of available samples for microscopy and PCR analysis. Removal of these duplicates and missing samples left 1839 RDT samples, 1710 BS1, 1650 BS2 and 1506 DBS samples ready for analysis (Table 1). Sixty (3.5%) slides with BS1 results could not be reviewed by the second reader (BS2) due to poor quality of smears and transportation challenges such as breakage.

**Table 1:** **Summary of all samples collected from 12 health facilities, in Iringa and Mufindi District Councils (DC)**

**District**	**Name of Health facility**	**Expected samples**	**Total RDT**	**Total BS1**	**Total BS2**	**Total DBS**
Iringa DC	Idodi Health Center	200	164	164	164	161
Iringa DC	Ifunda Dispensary	200	196	195	196	156
Iringa DC	Ilambilole Dispensary	200	185	180	170	156
Iringa DC	Kimande Health Center	200	195	158	194	166
Iringa DC	Tosamaganga Hospital	200	158	88	109	115
Iringa DC	Mlowa Dispensary	200	160	156	156	158
Mufindi DC	Igomaa Dispensary*	200	92	92	91	91
Mufindi DC	Kibao VA Health Center**	200	154	154	154	76
Mufindi DC	Mafinga Hospital	200	161	161	142	118
Mufindi DC	Malangali Health Center**	200	152	140	55	101
Mufindi DC	Sadani Health Center*	200	60	60	60	59
Mufindi DC	Usokami Health Center	200	162	162	159	149
Total		2400	1839	1710	1650	1506***

* Some samples had identical unique ID’s, hence removed from the analysis.

** Some samples were not sent for reference reading and/ or further analysis in Dar es Salaam.

*** DBS were not available from every sample.

Of the 1,839 samples tested by RDT, 120 (6.5%) were positive (Table 2). The local district microscopy (BS1) found 50 out of 1,710 (2.9%) positives; reference microscopy (BS2) found 42 out of 1,650 (2.5%) positives and qPCR1 found 64 out of 1,506 (4.2%) positives (Table 2). Fifteen out of the 64 qPCR1 positive samples had a parasite density of <200 parasites/μL and were not included in further comparative analysis with RDT and BS results. Malaria RDT had a higher overall positivity rate than microscopy and qPCR1, even when stratified by district (Table 2). The prevalence of malaria was found to be higher in Iringa District by both microscopy (BSsum) and qPCR1, but not by RDT which demonstrated higher positivity in Mufindi District (Table 2).

**Table 2:** **Number tested and number positive according to diagnostic test performed on specimens obtained from patients who attended study facilities - Iringa and Mufindi District Councils (DC)**

**Diagnostic test**	**Total**	**Iringa DC**	**Mufindi DC**	P value*
	**Tested Positive (%)**	**Tested Positive (%)**	**Tested Positive (%)**	
RDT	1839 120 (6.5)	1092 62 (5.7)	747 58 (7.7)	0.07
BS1	1710 50 (2.9)	941 34 (3.6)	769 16 (2.1)	0.11
BS2	1650 42 (2.5)	989 33 (3.3)	661 9 (1.4)	0.025
BS-sum	1555 34 (2.2)	933 29 (3.1)	622 5 (0.8)	0.002
qPCR1	1506 64 (4.2)	912 55 (6.0)	594 9 (1.5)	<0.001

*Chi-square test comparing district-level results.

Overall, the sensitivity of RDTs was 85.3% and 65.3% with microscopy (BSsum) and qPCR1 used as the gold standard, respectively. The predictive value positive of RDT was very low by both microscopy (BSsum, 31.5%) and qPCR1 (33.3%) (Table 3). RDT results were poorly correlated with both microscopy BS1 (K = 0.50), BS2 (K = 0.43) and qPCR1 (K = 0.45) results (Table 4). Microscopy results between the local district (BS1) and reference laboratories (BS2) showed 98.6% concordance (McNemar test) and a high inter-observer agreement (K = 0.75, p = 0.05) between them. Of the 300 DNA samples processed by qPCR2, 270 samples had satisfactory results. The qPCR1 and qPCR2 results had 88.2% concordance, but were poorly correlated (K = 0.32, p < 0.01).

**Table 3:** RDT sensitivity, specificity and predictive values* reference standard test; PPV = positive predictive value; NPP = negative predictive value

**Test combination (n)**	**Sensitivity**	**Specificity**	**PPV**	**NPV**
RDT/ BS-sum* (1555)	85.3%	95.8%	31.5%	99.7%
RDT/ qPCR1* (1506)	65.3%	95.6%	33.3%	98.8%

*Reference standard test; PPV= positive predictive value; NPP= negative predictive value.

**Table 4:** **Proportion of agreements between mRDT and microscopy and qPCR (McNemar comparison, Kappa statistic and correlation)**

**Tests combination**	**Observed agreement**	**Expected agreement**	**McNemar p-value**	Kappa statistic*	**Correlation**
RDT- BS1 (n = 1710)	95.6%	91.2%	<0.01	0.50	0.54
RDT – BS2 (n = 1650)	95.2%	91.5%	<0.01	0.43	0.48
RDT – qPCR1 (n = 1506)	94.9%	90.7%	<0.01	0.45	0.47
BS1 - BS2 (n = 1508)	98.6%	94.5%	=0.05	0.75	0.76

Kappa statistic*: p < 0.01.

Processing of BS1 took an average of 8 days (range = 1–30 days), while that of BS2 averaged 36 days (range = 16–90 days). Significant delays (sometimes up to months) were observed in processing and obtaining qPCR1 results, due to technical challenges including equipment breakdown, lack of in-country expertise to repair the apparatus necessitating shipment to South Africa and short shelf-life of reagents.

## Discussion

The aim of this study was to determine which of two methods, local qPCR (qPCR1) or microscopy (BS1), would provide optimal QA for RDTs in routine care. Neither of the two QA methods investigated correlated very well with RDT results, implying that these two tests (as performed here) may not be ideal for RDT QA or that inherent limitations of RDTs and/or of the RDT performer may cause inaccurate RDT test results. For example, RDTs are known to detect persistent HRP-2 antigen even after the successful treatment of malaria and clearance of asexual parasites [8,24,25]. The presence of circulating antinuclear antibodies such as in patients with rheumatoid arthritis, although a rare occurrence, has also been shown to give false positive RDT results [24,26]. In addition, studies in other malaria endemic areas have associated false positivity of RDTs with presence of gametocytes in blood [27] and acute schistosomiasis [28]. These other issues were not investigate in this study, but the higher positivity observed with RDT as compared to the other two tests can be partly explained by the detection of persisting HRP-2 antigen.

Other factors known to lead to false positivity by RDTs include inappropriate testing procedures (e.g., substituting test kit buffer solution with other liquids such as normal saline, distilled water, tap water or buffer from different kits/lots/ batches) [29]. Incorrect reading of RDT results by health workers during RDT testing is also known to influence test performance [3,30]. It is highly likely that health worker performance played a role in our findings because there is no other reason for RDT positivity rates to be higher in Mufindi district when all other malaria test results (BS1, BS2, qPCR1 and qPCR2) were lower in this district. Furthermore, community based surveys (through the Tanzania HIV and Malaria Indicator Surveys) and health facility data (as reported by District Health Information System 2) support higher transmission in Iringa district council than Mufindi district [31]. Although assessment of testing procedure during sample collection and supervisory visits showed very good adherence to the testing procedures it is also prone to observation bias (Hawthorne effect), hence a need to emphasize continued supportive supervision from health managers. False negative RDT results due to prozone effect have been reported [32]; however, there were no observed correlation between very high parasite density as detected by qPCR1 and microscopy with negative RDT results.

Studies have shown that the sensitivity of RDT compared to PCR and microscopy decline sharply at low parasite densities [9,33], therefore, care must be taken when comparing RDT results with molecular based diagnostic methods. The qPCR assay used in this study was selected on the basis that we would be able to quantify parasite densities and limit the comparison to RDT expected threshold of detection [8]. In this study, 15 qPCR1 positive samples had parasitemia of less than 200 parasites/μL and only two of these were detected as positive by RDT. Despite excluding these samples, we did not observe good correlation between RDT and qPCR1 unlike previous studies where high agreement between RDT and PCR [33-35] was reported.

Prompt responsiveness to problems with the quality of RDTs will ensure timely remedial strategies such as recalling defective RDT, providing refresher training to laboratory staff, or implementing closer supervision and follow-ups. This level of performance can only be practical where there is timely reporting of results to allow effective feed back into the system. Therefore, the timeliness of a QA test is an important consideration when selecting an appropriate QA test. The average microscopy processing time observed in this study was eight (8) days for district health facilities and 36 days at the reference laboratory. The lower end of this duration (i.e., 8 days) may be acceptable for use in routine QA, but improvement is needed to shorten this time to a fewer number of days.

The significant delays we observed in sample processing via qPCR1 and the fact that it was poorly correlated with qPCR2 suggest that this may not be a suitable method of QA in this setting. However, newer and simpler molecular assays that can overcome this limitation may provide good alternatives. This may include assays such as the loop mediated isothermal amplification (LAMP) assays [36] and Photo-induced Electron Transfer-PCR (PET-PCR) [37] that have shown to work in similar settings. The LAMP assay appears to be the most promising because of its simplicity, rapid turn around and sensitivity [38]. The potential of using used RDT devices as a source of parasite DNA for molecular analysis to monitor RDT performance in the field [39] should be further explored in different settings, keeping in mind the differences in detection limits of the two tests.

The importance of in-service training was clear in this study. All technicians were trained in microscopy before the study and the level of technical competence shown by the districts’ microscopists in this study, to stain and read slides, was encouraging. A good agreement between health facility and reference laboratory microscopy results was observed. This was a great improvement compared to previous result which demonstrated lack of competence in health facility microscopy [9]. The fact that microscopy is technically less challenging than qPCR and that the performance shown by the districts’ technicians was better correlated with the reference microscopists after training, demonstrates the practicality of using microscopy-based QA for monitoring performance of RDTs in routine care as previously indicated [40]. However, the lack of agreement with RDT in this study in addition to the long delays observed in obtaining microscopy results brings to question the use of microscope as a QA test for RDTs.

The new WHO guidelines for quality control of RDT in field use are focused on quarterly supportive supervision of RDT performers with assessment of the testing procedure in clinical settings [39]. This intense level of quality control at health facility level requires dedicated teams of supervisors who will visit the health facilities and perform the necessary assessment and provide the appropriate corrective action to address noted deficiencies. Poor supervision and adherence to procedures may result in poor quality RDT results, as previously reported in other parts of Tanzania [41]. The use of positive control wells which consist of lyophilized recombinant proteins (mainly HRP-2) which can be reconstituted in the field and used to test the correct performance of RDTs has been recommended [42]. An alternative to this is the proposed use of well-standardized *P. falciparum* - infected dried blood samples in a tube that can also be used as positive control samples for monitoring RDT performance [43].

## Conclusions

As highlighted in this study, finding an appropriate QA strategy for RDTs is challenging given the many possible factors that can compromise results from both the RDT and the selected QA test. Ensuring performer supervision in combination with the use of positive control samples to ascertain RDT performance is likely to provide the most practical and measureable QA approach for RDTs.
